# Links between soil microbial communities and plant traits in a species‐rich grassland under long‐term climate change

**DOI:** 10.1002/ece3.2700

**Published:** 2017-01-09

**Authors:** Emma J. Sayer, Anna E. Oliver, Jason D. Fridley, Andrew P. Askew, Robert T. E. Mills, J. Philip Grime

**Affiliations:** ^1^ Lancaster Environment CentreLancaster UniversityLancasterUK; ^2^ Smithsonian Tropical Research InstitutePanamaRepublic of Panama; ^3^ Department of Environment, Earth and EcosystemsThe Open UniversityMilton KeynesUK; ^4^ Centre for Ecology and HydrologyWallingfordUK; ^5^ Department of BiologySyracuse UniversitySyracuseNYUSA; ^6^ Department of Animal and Plant SciencesUniversity of SheffieldSheffieldUK

**Keywords:** Buxton, drought, grassland, resilience, resistance, soil bacteria, soil fungi, subordinate taxa

## Abstract

Climate change can influence soil microorganisms directly by altering their growth and activity but also indirectly via effects on the vegetation, which modifies the availability of resources. Direct impacts of climate change on soil microorganisms can occur rapidly, whereas indirect effects mediated by shifts in plant community composition are not immediately apparent and likely to increase over time. We used molecular fingerprinting of bacterial and fungal communities in the soil to investigate the effects of 17 years of temperature and rainfall manipulations in a species‐rich grassland near Buxton, UK. We compared shifts in microbial community structure to changes in plant species composition and key plant traits across 78 microsites within plots subjected to winter heating, rainfall supplementation, or summer drought. We observed marked shifts in soil fungal and bacterial community structure in response to chronic summer drought. Importantly, although dominant microbial taxa were largely unaffected by drought, there were substantial changes in the abundances of subordinate fungal and bacterial taxa. In contrast to short‐term studies that report high resistance of soil fungi to drought, we observed substantial losses of fungal taxa in the summer drought treatments. There was moderate concordance between soil microbial communities and plant species composition within microsites. Vector fitting of community‐weighted mean plant traits to ordinations of soil bacterial and fungal communities showed that shifts in soil microbial community structure were related to plant traits representing the quality of resources available to soil microorganisms: the construction cost of leaf material, foliar carbon‐to‐nitrogen ratios, and leaf dry matter content. Thus, our study provides evidence that climate change could affect soil microbial communities indirectly via changes in plant inputs and highlights the importance of considering long‐term climate change effects, especially in nutrient‐poor systems with slow‐growing vegetation.

## 1 Introduction

The extremely high diversity of soil microorganisms makes it difficult to establish links between individual microbial taxa and specific functions (Allison & Martiny, 2008). Shifts in community structure can give a first indication of when and how microbial adaptation will influence the rate of ecosystem processes (McGuire & Treseder, 2010), and hence, identifying the responses of microbial communities to change is an important first step to determining the functional consequences for ecosystems (Wallenstein & Hall, 2012; Zak, Pregitzer, Burton, Edwards, & Kellner, 2011).

Soil microbial communities carry out the bulk of decomposition (Swift, Heal, & Anderson, 1979) and catalyse many important processes that drive terrestrial carbon and nutrient cycling (Schlesinger, 1991). Changes in precipitation and temperature can affect soil microbial communities directly by altering their growth and activity but also indirectly via effects on the vegetation (Bardgett, Freeman, & Ostle, 2008). Plant‐mediated effects include changes in plant growth, biomass allocation, photosynthetic rate, litter quality, and water use efficiency, which are all likely to affect soil microbial communities (Gutknecht, Field, & Balser, 2012). At the same time, plant growth is also strongly influenced by the soil microbial community because plant nutrient requirements are largely met by the breakdown and mineralization of organic matter, which requires the combined activities of many different microorganisms (Burns et al., 2013). This reciprocal exchange of resources between plants and soil microbial communities underpins ecosystem function, succession, and recovery from disturbance (Reynolds et al. 2003) and is hence central to ecosystem responses to global change.

Contrary to the widely held view that high functional redundancy of microorganisms confers resilience and resistance of communities to perturbations, soil microbial communities are generally sensitive to change and not immediately resilient after disturbance (Allison & Martiny, 2008). Shifts in microbial community composition in response to disturbance arise primarily as a result of variation in the growth rates and resource‐use efficiencies of the constituent organisms, as well as their inherent resistance and acclimation capacities (Schimel, Balser, & Wallenstein, 2007). In general, soil fungi have higher C:N biomass stoichiometry, slower growth and turnover rates, and higher potential carbon use efficiency compared to bacteria (Waring, Averill, & Hawkes, 2013). The lower nutrient requirement of fungi and their ability to degrade recalcitrant plant litter gives them an advantage when resource quality is low, whereas rapid growth and turnover make bacteria better competitors for labile, high‐quality substrates (Waring et al., 2013). These general patterns of resource use and turnover support the widely held view that soil food webs dominated by fungi are more resistant to climate changes, whereas bacteria‐dominated systems are more resilient (De Vries et al., 2012a). Nonetheless, there are also substantial differences in the physiologies, adaptive capacities, and resource use of organisms within a given taxonomic group, which will shape community‐level responses to climate change.

Soil microbial communities that experience high natural variation in environmental conditions are likely to be dominated by generalist taxa with broad tolerances and resource use (Wallenstein & Hall, 2012). By contrast, taxa with specialist functions or high resource specificity are likely to be more sensitive to disturbance (Schimel, 1995). The responses of these subordinate taxa to climate changes may be particularly important for the functioning of nutrient‐poor systems because species‐rich plant assemblages have high chemical diversity (van der Heijden, Bardgett, & van Straalen, 2008), which requires greater microbial resource specificity and reduces functional redundancy (Waring et al., 2013). As soil microbes facilitate nutrient‐driven niche partitioning in plants (Reynolds & Haubensak, 2008), changes in microbial community structure or activity as a result of altered resource availability will feed back to affect plant nutrient availability.

Although there are multiple lines of evidence that climate change can rapidly affect soil microbial communities (Allison & Martiny 2008), long‐term experiments are required to assess indirect effects via changes in plant species composition, especially in systems with stress‐tolerant, slow‐growing vegetation. Importantly, differences in the resource use and adaptive capacities of generalist and specialist soil microorganisms also make plant‐mediated effects of climate change much harder to predict than the direct effects of changes to the abiotic environment. As a result, community‐level responses to long‐term chronic changes could differ substantially from the immediate responses to short‐term perturbations (Schimel et al., 2007).

We investigated long‐term changes in soil bacterial and fungal communities at the Buxton Climate Change Impacts Study (henceforth “Buxton”), where temperature and rainfall have been manipulated since 1993. Although the vegetation in this nutrient‐poor ancient grassland has proven remarkably resistant to change at the community level (Grime et al., 2008), there has been substantial small‐scale turnover in plant species composition within microsites (100 cm^2^) in response to summer drought and winter heating treatments (Fridley, Grime, Askew, Moser, & Stevens, 2011). Detailed existing data on plant species composition and plant traits in small‐scale microsites within the treatment plots (Fridley, Lynn, Grime, & Askew, 2016; Fridley et al., 2011) make this experiment an ideal platform to investigate potential links between plant and microbial responses to long‐term change. We hypothesized that long‐term climate manipulations would alter soil microbial communities and that the shifts in soil fungal and bacterial community structure would be related to changes in plant species composition via the quality of plant inputs to the soil.

## 2 Methods

The Buxton study was established in 1993 on calcareous grassland in Derbyshire, UK. Climate treatments are applied to 3‐m × 3‐m plots in five fully randomized blocks; a full description of the site and experimental design is given in Grime et al. (2000, 2008). The treatments sampled in the present study were: “heated” to 3°C above ambient temperature from November to April; “drought” in which rainfall is excluded during July and August; “watered” with water supplementation of 20% above the long‐term average from June to September; and nonmanipulated controls. Between 2006 and 2008, eight 10‐cm × 10‐cm microsites were established in each plot; the microsites were characterized by detailed measurements of surface soil pH (0–3 cm depth) and soil depth (Fridley et al., 2011), and all vascular plants in the microsites were surveyed in 2008 and 2012.

### 2.1 Sampling

Soil depth varies substantially across the study site, and as the most pronounced differences in plant species composition were observed between the shallowest and deepest microsites within plots (Fridley et al., 2011), we collected two soil samples from each of the two shallowest (0–7 cm soil depth) and the two deepest (>20 cm depth) microsites per plot in September 2011. To exclude immediate abiotic effects of the treatments, we sampled 1 month after the end of the annual drought and rainfall treatments but before the start of the winter warming treatment. We used a 1‐cm‐diameter punch corer to minimize disturbance to the vegetation and the sampling depth in all microsites was ≤10 cm. Two of the shallowest microsites were bare bedrock and no samples were collected; all other samples were composited to make one sample per microsite, making a total of 78 samples. All samples were transported to the laboratory on ice and frozen at −20°C on the same day.

### 2.2 Molecular analyses

To investigate the effects of climate treatments on soil microbial communities, we performed community fingerprinting using terminal restriction fragment length polymorphism (TRFLP) analysis of soil bacteria and fungi. This low‐cost high‐throughput method can perform as well as deep sequencing when investigating ecological patterns in microbial communities at local to regional scales (van Dorst et al., 2014) and provides qualitatively similar data for modeling community dynamics (Powell et al., 2015). DNA was extracted from 0.25 g of soil and resuspended following Griffiths, Whiteley, O'Donnell, and Bailey (2000) as described in Sayer et al. (2013). Briefly, we targeted the bacterial 16S rRNA gene using the primers 63F and 530R (Thomson, Ostle, & McNamara, 2010) and the fungal ITS region using the primers ITS1‐F and ITS4 (Klamer & Hedlund, 2004; Klamer, Roberts, Levine, Drake, & Garland, 2002). Forward primers were labeled at the 5′ end with 6FAM fluorescent dye, and PCR was conducted in 50 μl reaction volumes using 50 ng of template DNA. Amplicons were purified using PureLink PCR purification kits (Invitrogen, Paisley, UK) and digested using restriction endonuclease MspI for bacteria (Thomson et al., 2010) and Taq1 for fungi (Jasalavich, Ostrofsky, & Jellison, 2000; Singh, Dawson, Macdonald, & Buckland, 2009). Fragment analysis was performed using a 3730 DNA analyser (Applied Biosystems, CA, USA) and individual terminal restriction fragments (TRFs) were binned manually using Genemarker software (SoftGenetics, PA, USA). Prior to statistical analyses, the intensity of each TRF was converted to relative abundance based on the total intensity of all detected TRFs; for plot‐level analyses, we used the mean abundance of each TRF from the four microsites per plot. This approach provides a semiquantitative measure of abundance to assess differences in soil microbial community structure among sites but precludes measures of diversity (Bent, Pierson, & Forney, 2007) and excludes rare species (Woodcock, Curtis, Head, Lunn, & Sloan, 2006).

### 2.3 Data analyses

All statistical analyses were carried out in R version 3.2.3 (R Core Team 2014), and all multivariate analyses were performed using the vegan (Oksanen et al., 2011) package. As soil depth and pH within microsites were inversely correlated, we used the first axis scores from a principal components analysis of multiple soil depth and pH measurements to characterize each microsite (Fridley et al., 2011; henceforth “microsite scores”). For each microsite, we also included plant species data from Fridley et al. (2011, 2016) representing eight 10‐cm × 10‐cm quadrats within each 3 × 3‐m plot and community‐weighted plant trait data from Fridley et al. (2016), representing the quality and quantity of resources available to soil microorganisms: specific leaf area (measured as fresh leaf area per gram dry mass); maximum photosynthetic capacity (measured from light curves); leaf construction cost (in gram glucose per gram leaf following Heberling & Fridley, 2013); leaf dry matter content (dry‐to‐fresh mass ratio); and leaf C:N ratio. Community‐weighted trait values were calculated by taking the weighted average of trait values of those species present in a given microsite from their abundances, using visual cover classes (0–4, 5–24, 25–49, 50–74, 75%+; Fridley et al., 2016).

The effects of climate treatments on soil fungal and bacterial community composition at the plot level were examined by permutational multivariate analysis of variance (PerMANOVA; adonis function) after testing for homogeneity of dispersions among treatments (betadisper function); models were tested with 9,999 permutations constrained within blocks of replicate plots (permutest function). We used nonmetric multidimensional scaling (NMDS) based on Bray–Curtis dissimilarities to represent shifts in soil microbial communities (metaMDS function); stable solutions with stress scores <0.2 and *r* ^2^ > .95 were used for subsequent analyses, resulting in a two‐dimensional solution for bacteria and a three‐dimensional solution for fungi. We then used vector fitting to the NMDS ordinations (envfit function) to determine the effects of microsite, climate treatments, extracellular enzyme activities, and key plant traits; significance values were generated with 9,999 random permutations stratified within experimental blocks.

Pairwise concordance between plant species composition and soil fungal or bacterial communities in microsites within each treatment was investigated using Procrustes rotation (Procrustes function) based on the most stable NMDS solutions for all three communities; the Procrustes statistic was tested with 9,999 permutations (protest function). As no vegetation survey was conducted in the soil microbial sampling year (2011), we first compared plant species composition from the 2008 and 2012 surveys and then performed separate comparisons for each year. Plant species composition was highly correlated between survey years (Procustes correlation: *m* ^2^ = .58, *r* ^2^ = .65, *p* < .001), and we found the same degree of concordance between plant species and microbial community composition regardless of survey year. We therefore used the 2012 vegetation data for all subsequent analyses.

To differentiate the responses of dominant and subordinate fungal and bacterial taxa, we performed all ordinations with and without the most abundant TRFs. We used conservative cutoff points so that <10% of all taxa were considered abundant. Consequently, dominant taxa were defined as those with total relative TRF abundance >0.75% across all plots for fungi and >1% for bacteria; all other TRFs were considered as subordinate taxa.

## 3 Results

**Figure 1:**
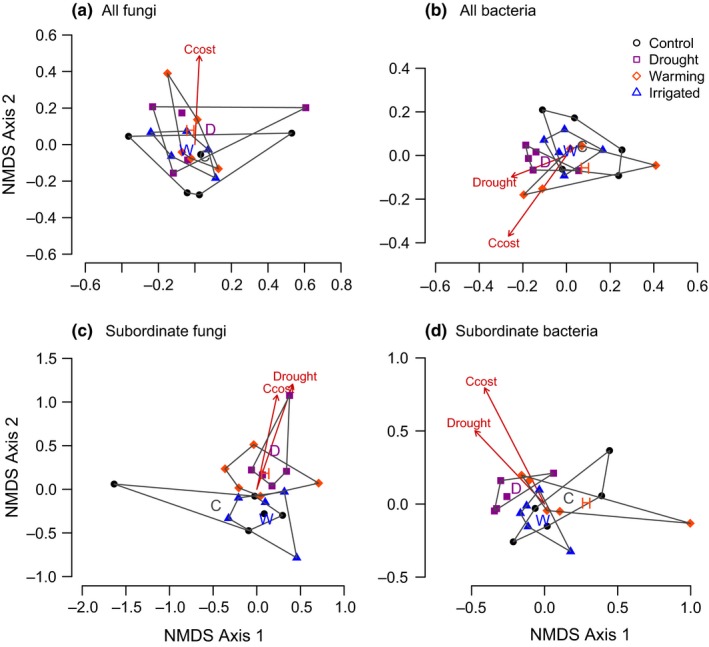
NMDS representation of (a) soil fungal communities, (b) soil bacterial communities, (c) subordinate fungal taxa, and (d) subordinate bacterial taxa in grassland plots subjected to long‐term climate treatments; ordinations were based on Bray–Curtis dissimilarities and hulls envelope all plots within a treatment, where “C” is control, “H” is heated, “D” is drought, and “W” is watered; significant correlations (*p* < .05) between ordination axes and treatments or community‐weighted plant traits are shown as arrows, where “Ccost” is the construction cost of plant material

Seventeen years of climate treatments resulted in divergent soil fungal and bacterial communities at the whole‐plot level (PerMANOVA *F*_3,19_ = 1.00, *p* = .028 and *F*_3,19_ = 1.47, *p* = .01 for fungi and bacteria, respectively). We identified a total of 230 fungal and 112 bacterial taxa across all microsites; the dominant taxa (22 fungal and 19 bacterial taxa) were present in all treatments at similar relative abundances (Fig. S1), but we observed pronounced differences in subordinate taxa (fungi: *F*_3,19_ = 1.20, *p* = .003; bacteria: *F*_3,19_ = 1.53, *p* = .008; Figures 1c,d, S2, and S3).

Vector fitting to NMDS ordinations of plot‐level data revealed that the heating and watered treatments had little or no effect, but there were substantial changes in soil microbial communities in the drought plots. There was only a minor effect of the summer drought treatment on the whole soil fungal community (*r* ^2^ = .12, *p* = .073; Figure 1a) but subordinate fungal taxa were significantly affected by drought (*r* ^2^ = .27, *p* = .044; Figure 1c) and 66 of the 208 subordinate fungal taxa were entirely absent from the drought treatments. Drought also altered bacterial community structure (*r* ^2^ = .2, *p* = .02; Figure 1b) but subordinate taxa were not disproportionately affected (*r* ^2^ = .25, *p* = .033; Figure 1d) and only six of the 93 subordinate bacterial taxa were entirely absent from the drought plots.

**Figure 2:**
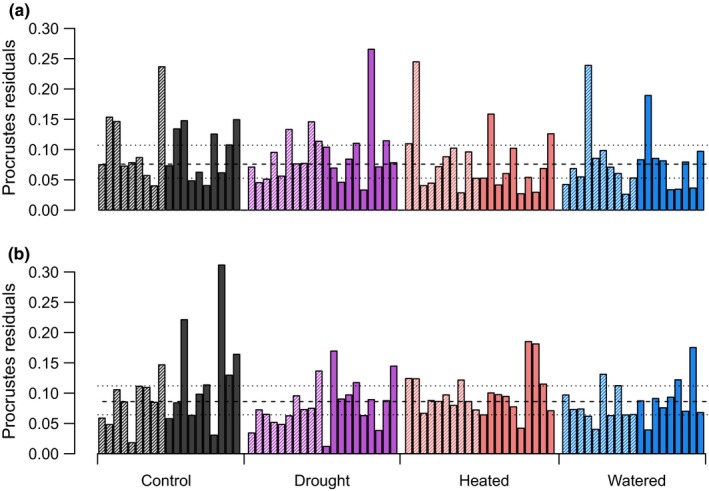
Residuals of Procrustes rotations (unitless) showing the associations between NMDS solutions of plant species composition and (a) soil fungal communities or (b) soil bacterial communities for each microsite within the Buxton climate treatments, where pale shaded bars denote shallow microsites and solid bars denote deep microsites; median (dashed line) and upper and lower quartiles (dotted lines) are shown; large residuals indicate individual microsites with a weak concordance between plant and microbial communities

There was moderate concordance between plant species and soil microbial community composition in microsites (Procrustes correlation: *m* ^2^ = .81, *r* ^2^ = .44, *p* = .001 for fungi and *m* ^2^ = .84, *r* ^2^ = .4, *p* = .001 for bacteria; Figure 2); the strength of the relationships decreased slightly when dominant taxa were excluded (*m* ^2^ = .88, *r* ^2^ = .34 and *m* ^2^ = .86, *r* ^2^ = .37 for fungi and bacteria, respectively).

**Figure 3:**
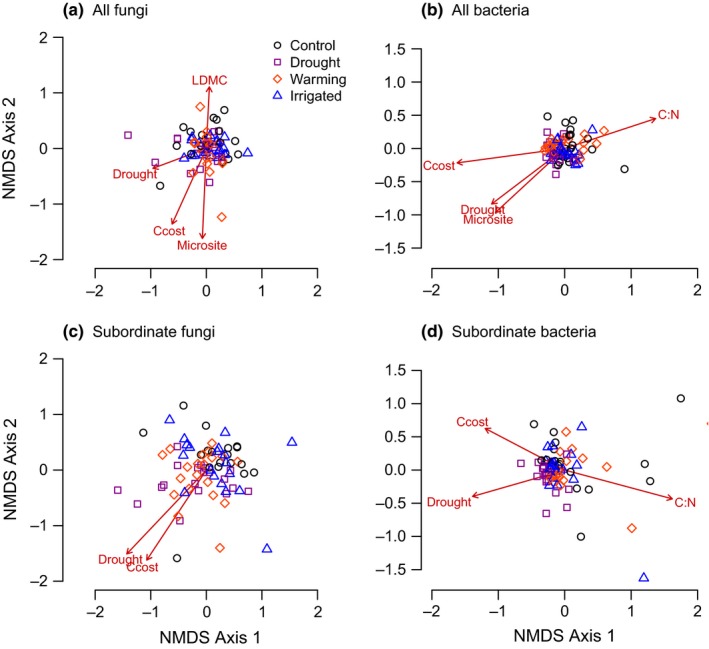
NMDS representation of (a**)** the soil fungal community, (b) the soil bacterial community, (c) subordinate fungal taxa, and (d**)** subordinate bacterial taxa in microsites within the Buxton climate treatments; ordinations were based on Bray–Curtis dissimilarities and significant correlations of community‐weighted plant functional traits and environmental variables with ordination axes are shown as arrows, where “Msite” is a microsite score based on multiple measurements of soil depth and pH, “Ccost” is the construction cost of plant material, “C:N” is the carbon‐to‐nitrogen ratio of plant material, and “LDMC” is leaf dry matter content

Vector fitting of microsite scores and plant trait data to NMDS ordinations of soil microbial taxa revealed a significant correlation between microsite characteristics and soil fungal (*r* ^2^ = .16, *p* = .003) and bacterial community structure (*r* ^2^ = .15, *p* = .003), but individual plant traits explained a similar or greater amount of variation in soil microbial communities among microsites: Leaf construction cost was the best predictor of shifts in community structure for both fungi (*r* ^2^ = .14, *p* = .004) and bacteria (*r* ^2^ = .20, *p* = .001). Soil fungal community structure was also related to leaf dry matter content (*r* ^2^ = .08, *p* = .03; Figure 3a), whereas bacterial community structure was related to plant C:N ratios (*r* ^2^ = .15, *p* = .009; Figure 3b). Changes in the relative abundances of subordinate microbial taxa were also associated with the construction cost of plant material (*r* ^2^ = .1, *p* = .025 and *r* ^2^ = .09, *p* = .018 for fungi and bacteria, respectively; Figure 3c), and shifts in subordinate bacterial taxa were also related to the C:N ratio of plant material (*r* ^2^ = .13, *p* = .037; Figure 3d).

## 4 Discussion

Long‐term climate treatments at Buxton modified the communities of bacteria and fungi in the soil. Whereas the relative abundances of dominant microorganisms were similar among treatments, we observed changes in subordinate fungal and bacterial taxa. We found evidence for potential links between the observed changes in soil microbial community structure and specific traits of the plant communities within microsites.

### 4.1 Climate treatment effects on soil microbial community structure

In contrast to previous studies, we found a strong effect of drought on soil fungal community structure in microsites within the climate treatments, which was largely a result of changes in the relative abundances of subordinate fungi (Figures 1c and S2). This was unexpected because fungi are widely regarded as drought‐tolerant (Harris, 1981; Schimel et al., 2007) and previous studies have reported high resistance of soil fungi to drought treatments (De Vries et al., 2012a; Fuchslueger, Bahn, Fritz, Hasibeder, & Richter, 2014; Yuste et al., 2011). We propose two possible explanations for this apparent discrepancy:

1. Changes in the abundances of fungal taxa may be more important and more readily apparent in grasslands such as Buxton, because fungal decomposers are more important in systems with low soil fertility and slow‐growing perennial plant species (De Vries et al., 2012b). Conversely, many of the studies reporting high resistance of soil fungi to drought were conducted in productive grasslands with bacteria‐dominated soil food webs (e.g. De Vries et al., 2012a,b; Fuchslueger et al., 2014).
2. To our knowledge, our study is the first to distinguish the responses of dominant and subordinate microbial taxa to long‐term climate treatments. On the one hand, the dominant taxa were largely unaffected by drought, which supports the hypothesis that they are likely to be generalists with a broad range of tolerances (Wallenstein & Hall, 2012). On the other hand, 32% of all subordinate fungal taxa were entirely absent from the drought treatments. As subordinate taxa represent only a small proportion of the total fungal community, these changes would go undetected in studies using indiscriminate or low‐resolution methods such as microbial biomass or lipid biomarkers, because those measurements would primarily reflect changes in most abundant taxa.

We used general fungal primers that do not specifically target mycorrhizal fungi, and consequently, the observed shifts in the drought treatments will mainly reflect differences in the abundances of decomposer and pathogenic fungi (Klamer & Hedlund, 2004; Sayer et al., 2013). Nonetheless, a previous study at Buxton showed that the drought treatments reduced the density of extraradical mycorrhizal hyphae in the soil (Staddon et al., 2003), which could also contribute to the shifts in fungal community structure in our study.

Drought also altered bacterial community structure in the soil but subordinate taxa were not disproportionately affected (Figure 1b,d) and only six taxa were entirely absent from the drought plots. As the treatments were applied during two summer months each year, the high resilience of bacteria to short‐term “pulse” disturbances (Shade et al., 2012) could allow most soil bacterial taxa to persist in all treatments and microsites. Furthermore, dormant organisms, which are included in our community fingerprints (Rastogi & Sani, 2011), can persist under unfavorable conditions, and rapid growth rates would allow dormant bacteria to recover rapidly after the end of a treatment period (Shade et al., 2012).

### 4.2 Links between plant functional traits and soil microbial responses to change

Theoretically, the soil microbial communities at Buxton should be adapted to drought because they naturally experience high variation in soil moisture (Schimel et al., 2007; Wallenstein & Hall, 2012). Nonetheless, 17 years of chronic summer drought altered soil microbial communities and the relationships between soil microbial community structure, plant species composition, and plant traits within the microsites at Buxton provide evidence to support our hypothesis for indirect effects of climate change on soil microbial community structure via plant inputs. The climate treatments at Buxton have resulted in distinct plant communities in microsites within the drought and heated plots (Fridley et al., 2011). A recent study of community‐weighted plant traits demonstrated greater investment in leaf material by slow‐growing, stress‐tolerant plant species in the drought plots, whereas the plant traits in the heated plots reflect greater productivity of more competitive species (Fridley et al., 2016). We hypothesized that shifts in plant traits representing the quality and quantity of resources available to microorganisms could explain some of the observed changes in soil microbial communities.

Changes in soil microbial communities in the drought plots were related to the high leaf construction cost of slow‐growing, stress‐tolerant vegetation (Figures 1 and 3; Fridley et al., 2011). Shifts in the relative abundances of microbial taxa could therefore reflect increased abundance of organisms that are able to degrade recalcitrant plant material, with concomitant declines in taxa dependent on labile, nutrient‐rich plant material. Shifts in the soil fungal community were also related to leaf dry matter content, whereas changes in soil bacteria were related to differences in plant C:N ratios (Figure 3). These relationships indicate that shifts in the abundances of specific taxa could be linked to decomposition processes because fungal decomposers are specialized in degrading tough, nutrient‐poor carbon sources, whereas many soil bacterial groups preferentially use more labile plant material and have higher nutrient requirements (De Boer, Folman, Summerbell, & Boddy, 2005).

Changes in the relative abundance of subordinate taxa were also related to functional traits associated with the quality of plant material (Figure 3b,d), but intriguingly, the concordance between plant and microbial communities decreased when dominant taxa were excluded. This suggests that the observed links between microbial community structure and vegetation within microsites could be associated with resource quality, rather than with particular plant species. Species‐rich plant assemblages have high chemical diversity, which requires greater microbial resource specificity and reduces functional redundancy (van der Heijden et al., 2008). The substantial losses of subordinate fungal taxa in the drought plots could therefore indicate greater sensitivity of specialists with high resource specificity or competitive exclusion by organisms that have benefitted from the changes in plant inputs. As the relative abundances of the dominant microbial taxa remained largely unchanged (Fig. S1), the shifts in soil microbial community structure among climate treatments were largely driven by the responses of subordinate taxa and we propose that changes in plant inputs within microsites represent a plausible mechanism for the shifts in subordinate soil microbial taxa.

As the aim of our study was to investigate the possibility of indirect, biotic effects of climate changes, we collected soil samples when no treatments were actively being applied; this precludes analysis of seasonal changes in microbial communities but ensured comparable soil temperature and soil water content among treatments (Fridley et al., 2016). We found evidence of links between plant traits and soil microbial communities despite the breadth of microbial functional groups included in our community fingerprints; the seemingly small effect sizes in our study are unsurprising, given the high microbial diversity and the potential influence of numerous soil physical and chemical properties. Soil pH in particular has an overriding effect on soil bacteria (Fierer, Bradford, & Jackson, 2007; Griffiths et al., 2011), and it is conceivable that some of the observed differences in bacterial community structure are a result of lower soil pH in the drought plots and deep microsites (Fridley et al., 2011) or a direct effect of differences in soil depth. Nevertheless, plant C:N ratios explained a greater proportion of the variation in soil bacteria compared to microsite characteristics (soil depth and pH) and the carbon construction costs of plant material explained an equivalent amount of variation in both bacterial and fungal community structures. In addition, the effect of microsite was weaker or entirely absent when dominant microbial taxa were excluded, whereas the effect of drought and the relationship with plant functional traits remained (Figure 3). This suggests that subordinate taxa may be more sensitive to changes in resource quality than soil depth or pH. Hence, we suggest that the concordance between plant species composition and microbial community structure in our study provides evidence for plant‐mediated effects of climate change on soil microbial communities and the links to specific plant traits suggest that decomposition processes may play an important role in concerted above‐ and belowground responses to long‐term climate change (McGuire & Treseder, 2010).

## 5 Conclusions

Our study highlights the importance of considering long‐term and indirect effects of climate changes on soil microbial communities, especially in nutrient‐poor systems with slow‐growing vegetation. Whereas climate changes can rapidly modify the abiotic environment, comprehensive shifts in community‐level plant functional traits are likely to become more relevant and more apparent over time. The unexpected drought response of soil fungi and the links between microbial communities and key plant functional traits in our study suggest that microbial resistance or acclimation to direct climate change effects could be subsumed by altered plant species composition in the long run, with as yet unknown consequences for ecosystem function.

## Conflict of interest

The authors have no conflict of interest to declare.

## Data accessibility

All original data used in this paper will be archived in Dryad and made publicly accessible upon publication; previously published data on plant species composition and plant traits, which were used in the analyses, are referenced in the text.
