# Supplementary material for: Links between soil microbial communities and plant traits in a species‐rich grassland under long‐term climate change
Source: Ecol Evol. 2017 Jan 9;7(3):855–62. doi: 10.1002/ece3.2700 (PMC5288249; doi:10.1002/ece3.2700)

**Appendix**

**Supplementary Figure S1.** Relative abundances of dominant **a)** fungal and **b)** bacterial terminal restriction fragments (TRFs) in grassland soil in the Buxton climate treatments, given as a proportion of the total relative abundance of all TRFs across all plots; bar labels indicate restriction sites in base-pair length (nt).

| **a)** 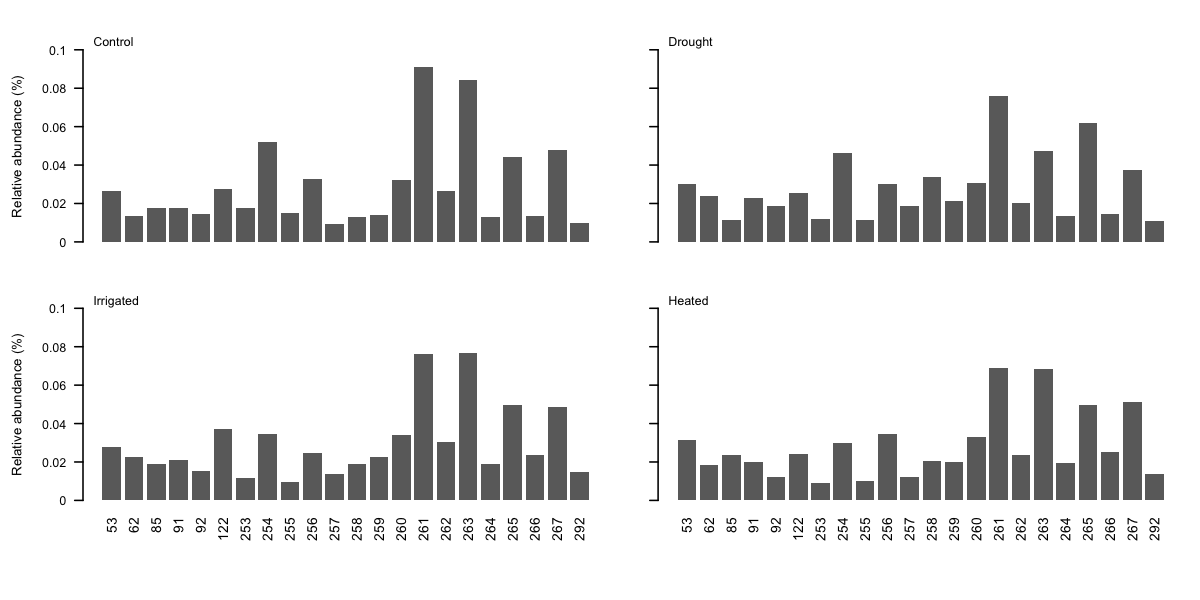 |
| --- |
| **b)** 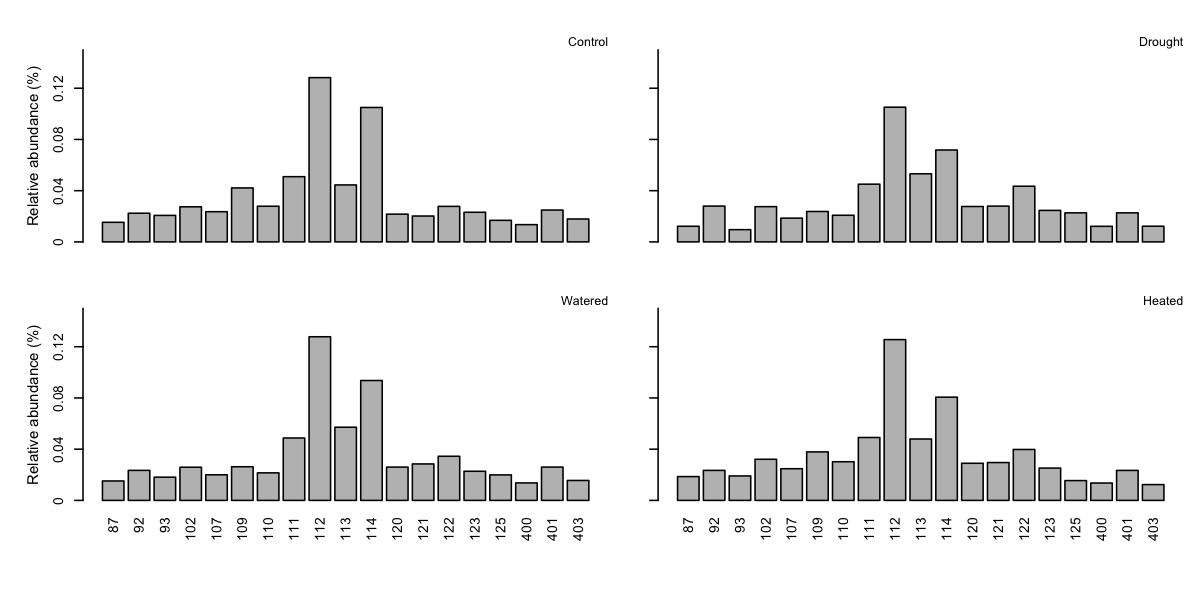 |

**Supplementary Figure S2.** Relative abundances of subordinate soil fungal taxa (terminal restriction fragments; TRFs) in grassland soil in the Buxton climate treatments, given as a proportion of the total abundance of all TRFs across all plots.


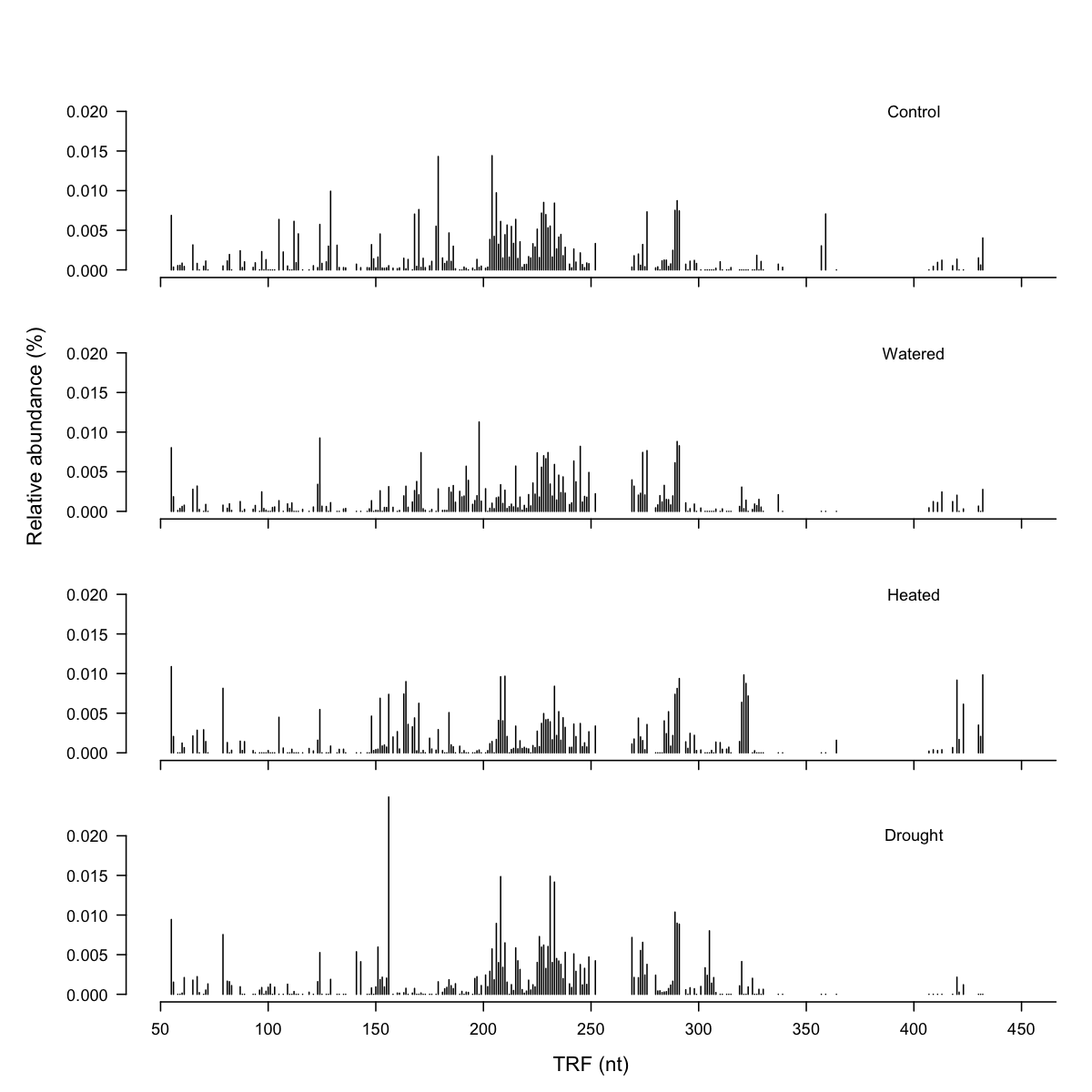


**Supplementary Figure S3.** Relative abundances of subordinate soil bacterial taxa (terminal restriction fragments; TRFS) in grassland soil in the Buxton climate treatments, given as a proportion of the total abundance of all TRFs across all plots.


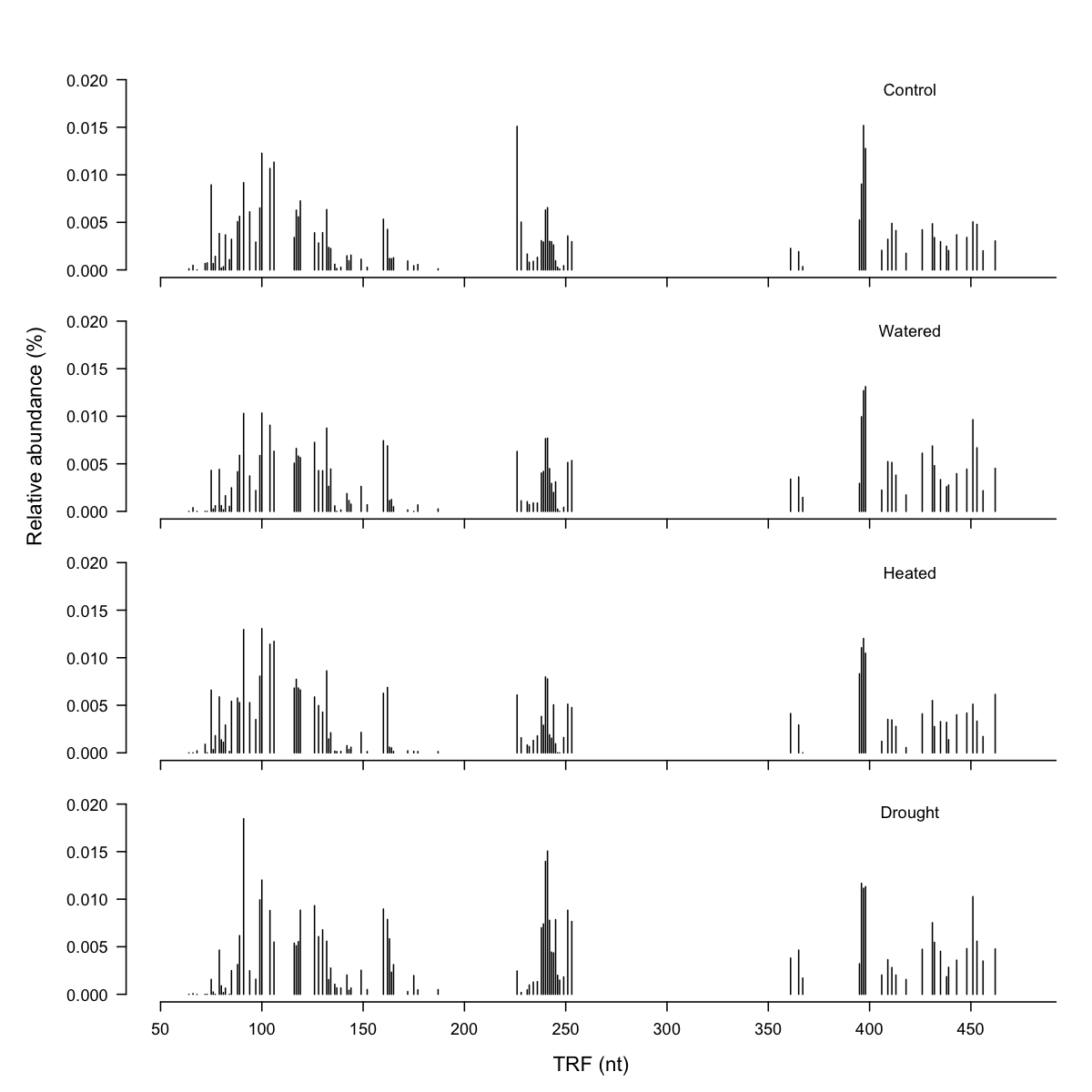

Supplement: Supplementary file 1 [file ECE3-7-855-s001.docx]
